# Associations of the hypertension-related single nucleotide polymorphism rs11191548 with high-density lipoprotein cholesterol and leptin in Chinese children

**DOI:** 10.1186/s12881-018-0523-y

**Published:** 2018-01-16

**Authors:** Lijun Wu, Liwang Gao, Xiaoyuan Zhao, Meixian Zhang, Jianxin Wu, Jie Mi

**Affiliations:** 1 Department of Epidemiology, Capital Institute of Pediatrics, No. 2 Yabao Road, Chaoyang District, Beijing, 100020 China; 2 Department of Biochemistry, Capital Institute of Pediatrics, No. 2 Yabao Road, Chaoyang District, Beijing, 100020 China

**Keywords:** Leptin, High-density lipoprotein cholesterol, *CYP17A1*, Hypertension-related single nucleotide polymorphism

## Abstract

**Background:**

The genome-wide association study has founded hypertension-related single nucleotide polymorphism (SNP) rs11191548 near *CYP17A1* encoding a key enzyme involved in steroid metabolism, but the molecular mechanisms are not understood and the associations of the SNP with hypertension-related traits are not fully described, especially in children. The aim of the present study is to investigate the associations between the SNP and two hypertension-related traits, lipids and leptin.

**Methods:**

We genotyped the SNP in Beijing Child and Adolescent Metabolic Syndrome (BCAMS) study. A total of 3503 children participated in the study.

**Results:**

The SNP rs11191548 was significantly associated with high-density lipoprotein cholesterol (HDL) (*P* = 0.014 and 0.028, respectively) and leptin (*P* = 0.011 and 0.026, respectively) under an additive model after adjustment for age, gender, and systolic blood pressure (SBP) or diastolic blood pressure (DBP). There was a statistically significant association of rs11191548 with high leptin after adjustment for age, gender, and SBP or DBP. The *P-values* remain significant after correction for multiple testing.

**Conclusions:**

We demonstrate for the first time that the SNP rs11191548 near *CYP17A1* is associated with HDL and leptin in Chinese children. These novel findings provide important evidence that HDL and leptin maybe possibly mediate the process of CYP17A1 involved in hypertension.

**Electronic supplementary material:**

The online version of this article (10.1186/s12881-018-0523-y) contains supplementary material, which is available to authorized users.

## Background

In recent years, the prevalence of hypertension has been increasing in most parts of the world, and hypertension is a major threat to public health [1]. Childhood hypertension is a predictor of adult hypertension and cardiovascular disease [2, 3].

Previously, multiple single nucleotide polymorphisms (SNPs) related to hypertension have been identified by genome-wide association studies [4–6]. Among those identified SNPs, the SNP rs11191548, located near the 3′ noncoding region of the gene *CYP17A1* encoding the cytochrome P450 enzyme CYP17A1as a key enzyme involved in steroid metabolism, showed a significant association with hypertension in European adults, Japanese and Chinese adults, and Chinese children [6–9]. However, the molecular mechanisms are not understood and the associations of the SNP with other hypertension -related traits are not fully described.

Dyslipidemia are recognized as a strong predictor of cardiovascular disease and several studies have suggested a positive correlation between dyslipidemia and hypertension [10, 11]. Leptin, an adipocytokine produced by adipose tissue, is associated with dyslipidemia [12], and recent study has demonstrated that leptin contributes to hypertension through upregulation of central renin–angiotensin system and proinflammatory cytokines [13].

There has been no evidence that the SNP rs11191548 near *CYP17A1* is associated with lipids and leptin. We investigated the associations between the SNP rs11191548 with lipids and leptin in the cohort. We genotyped the SNP in Chinese children who were participated in the population-based Beijing Child and Adolescent Metabolic Syndrome (BCAMS) study. The present study attempts to provide an analysis of epidemiological and genetic data towards the possible mechanism of the role of *CYP17A1* in hypertension.

## Methods

### Study population

Subjects were recruited from a cross-sectional population-based survey termed the BCAMS study in 2004. The survey included a questionnaire, anthropometric measurement, and medical examination in a representative sample (*n* = 19,593, 50% boys) of children in Beijing aged 6–18 years. Anthropometric measurements included weight, height, waist circumference, and fat mass percentage. Within this large group of children, 1045 children with elevating blood pressure (including pre-hypertension and hypertension) and 2458 children with normal blood pressure were randomly recruited and diagnosed by using blood pressure reference cutoffs for Chinese children and adolescents [14]. Venipuncture blood samples were collected for genotyping. The BCAMS study was approved by the ethics committees of Capital Institute of Pediatrics. We obtained written informed consent from parents or guardians.

### Measurement of biochemical analyses and genotyping

Total cholesterol (TC), low-density lipoprotein cholesterol (LDL), high-density lipoprotein cholesterol (HDL), triglycerides (TG), were analyzed by an automatic biochemical analyzer (Hitachi 7060) using a kit assay (SEKISUI medical technology Ltd., Tokyo, Japan). The levels of adipocytokines were measured by ELISA techniques [15]. Genomic DNA was isolated from peripheral white blood cells using the salt fractionation method. Genotyping of rs11191548 was conducted using the TaqMan Allelic Discrimination Assay with the GeneAmp 7900 Sequence Detection System (Applied Biosystems, Foster City, CA, USA), with the TaqMan probes (C_31979323_10). The genotyping call rate for the SNP was 97.9%. We sent 30 samples to direct sequencing and observed 100% concordance between two genotyping methods. We also repeated 70 samples randomly for the SNP to validate the accuracy of genotyping and observed 100% concordance between the results of the two tests.

### Statistical analysis

Categorical variables were presented as percentages and continuous variables were presented as mean ± standard deviation (SD). Hardy-Weinberg equilibrium was assessed using the chi-square test. Adjusted odds ratios (ORs) for high leptin were performed by logistic regression with genotypes, age, gender, and systolic blood pressure (SBP) or diastolic blood pressure (DBP) as the independent variables. The data were analysed using SPSS statistical software. *P* < 0.05 was used to indicate statistically significant differences. False discovery rate (FDR) approach was used to correct for multiple testing. In brief, the stringent *p-value* was considered statistically significant only if it was less than 0.05. Power calculation was performed using Quanto software according to the assumed effect size and allele frequency (http://hydra.usc.edu/gxe/).

## Results

The basic characteristics of the study participants are summarized in Table S1 (Additional file 1). We genotyped the SNP rs11191548 near *CYP17A1* in the cohort, and the genotype of the SNP was tested to be in Hardy–Weinberg equilibrium (*P* = 0.795). The minor allele frequency (MAF) of the SNP was 0.277 in the cohort. The associations of the SNP rs11191548 with HDL and leptin are shown in Table 1. As the SNP was associated with blood pressure, we also adjusted SBP or DBP besides age and gender. The SNP rs11191548 was significantly associated with HDL under an additive model (*P* = 0.014 and 0.028, respectively) after adjustment for age, gender, and SBP or DBP. The SNP was also significantly associated with leptin under an additive model (*P* = 0.011 and 0.026, respectively) after adjustment for age, gender, and SBP or DBP.

**Table 1:** Associations of rs11191548 with HDL and leptin

Additive model		HDL							Leptin						
	N	Mean ± SD	*P-value* ^a^	Power	*P-value* ^b^	Power	*P-value* ^c^	Power	Mean ± SD	*P-value* ^a^	Power	*P-value* ^b^	Power	*P-value* ^c^	Power
CC	260	1.36 ± 0.32							10.71 ± 12.72						
CT	1380	1.40 ± 0.32							10.42 ± 11.63						
TT	1791	1.41 ± 0.32	0.043	0.552	0.014^d^	0.691	0.028^d^	0.600	9.70 ± 11.14	0.051	0.558	0.011	0.745	0.026	0.646

*HDL* high-density lipoprotein cholesterol, *SD* standard deviation

^a^ Adjusted for age and gender, ^b^ Adjusted for age, gender and SBP, ^c^ Adjusted for age, gender and DBP, ^d^ *P-value* remains significant after FDR test is applied

Table 2 shows the association of rs11191548 with high leptin defined as leptin≥ the 75 percentile of the participant with same age and gender. The SNP rs11191548 was significantly associated with high leptin under an additive model after adjustment for age, gender, and SBP or DBP. The *P-values* remain significant after correction for multiple testing.

**Table 2:** Association of rs11191548 with high leptin under the additive model

Factors	Adjusted for age and gender				Adjusted for age, gender and SBP				Adjusted for age, gender and DBP			
	OR	95% CI	*P*-value	Power	OR	95% CI	*P*-value	Power	OR	95% CI	*P-value*	Power
High leptin	0.874	0.782–0.978	0.019^a^	0.700	0.841	0.749–0.945	0.004^a^	0.889	0.855	0.762–0.960	0.008^a^	0.822

*SBP* systolic blood pressure, *DBP* diastolic blood pressure, *OR* odds ratio, *CI* confidence interval

^a^ *P-value* remains significant after FDR test is applied

## Discussion

*CYP17A1* encodes the cytochrome P450 enzyme CYP17A1 and has a key role in the biosynthesis of steroid hormones [16]. CYP17A1 deficiency caused by a mutation in the gene usually results severe hypertension and hypokalemia in males [17]. The SNP rs11191548 near *CYP17A1* showed a significant association with hypertension [6–9], but the molecular mechanisms are not understood.

In this study, we examined the SNP rs11191548 near *CYP17A1* with lipids and leptin in Chinese children. Our results indicated that the SNP rs11191548 was significantly associated with HDL and leptin after adjustment for age, gender, and SBP or DBP, and there was statistically significant association of the SNP rs11191548 with high leptin under an additive model adjusted for age, gender, and SBP or DBP, after correction for multiple testing. No significant associations of the SNP with TC, TG and LDL were found in the population (data not shown). Studies with greater sample size are needed to confirm these associations.

This study may not provide direct evidence that the expression of *CYP17A1* influences hypertension because the lack of gene expression data, but our study demonstrated the associations of the SNP rs11191548 near *CYP17A1* with HDL and leptin that contribute to hypertension.

## Conclusion

We demonstrate for the first time that the SNP rs11191548 near *CYP17A1* is associated with HDL and leptin in Chinese children. These novel findings provide important evidence that HDL and leptin maybe possibly mediate the process of CYP17A1 involved in hypertension.

## Additional file

Additional file 1: TAble S1. Basic characteristics of study participants (DOC 42 kb)

## Abbreviations

DBP: Diastolic blood pressure
FDR: False discovery rate
HDL: High-density lipoprotein cholesterol
LDL: Low-density lipoprotein cholesterol
ORs: Odds ratios
SBP: Systolic blood pressure
SD: Standard deviation
SNPs: Single nucleotide polymorphisms
TC: Total cholesterol
TG: Triglycerides
