# Supplementary material for: Associations of the hypertension-related single nucleotide polymorphism rs11191548 with high-density lipoprotein cholesterol and leptin in Chinese children
Source: BMC Med Genet. 2018 Jan 16;19:9. doi: 10.1186/s12881-018-0523-y (PMC5771196; doi:10.1186/s12881-018-0523-y)
Supplement: Additional file 1: TAble S1. — Basic characteristics of study participants (DOC 42 kb) [file 12881_2018_523_MOESM1_ESM.doc]

**Supplementary Table 1** Basic characteristics of study participants

| Characteristics | All |
| --- | --- |
| N | 3503 |
| Male (%) | 50.8 |
| Age (years) | 12.4±3.1 |
| BMI (kg m^-2^) | 21.9±4.9 |
| Systolic blood pressure (mmHg) | 108±14 |
| Diastolic blood pressure (mmHg) | 68±10 |
| Hypertensions (%) | 17.7 |
| Total cholesterol (mmol L^-1^) | 4.1±0.8 |
| LDL (mmol L^-1^) | 2.6±0.8 |
| HDL (mmol L^-1^) | 1.4±0.3 |
| Triglycerides (mmol L^-1^) | 1.0±0.6 |
| Leptin (µg L^-1^) | 10.0±11.4 |
| Adiponectin (mg L^-1^) | 12.8±7.4 |
| Resistin (µg L^-1^) | 18.4±24.1 |

Data are presented as mean ±standard deviation, or percentages of subjects, as appropriate.

*BMI* body mass index, *HDL* high-density lipoprotein cholesterol, *LDL* low-density lipoprotein cholesterol

Hypertension was diagnosed using blood pressure reference cutoffs for Chinese children and adolescents.^1^

^1^ Xi B, Zong X, Kelishadi R, Hong YM, Khadilkar A, et al. Establishing International Blood Pressure References Among Nonoverweight Children and Adolescents Aged 6 to 17 Years. Circulation. 2016;133:398-408.
